# Hyperemesis gravidarum in the Medical Birth Registry of Norway – a validity study

**DOI:** 10.1186/1471-2393-12-115

**Published:** 2012-10-24

**Authors:** Åse Vikanes, Per Magnus, Siri Vangen, Sølvi Lomsdal, Andrej M Grjibovski

**Affiliations:** 1 Division of Epidemiology, Norwegian Institute of Public Health, Oslo, Norway; 2 Department of Obstetrics and Gynaecology and Medical Faculty Division, Akershus University Hospital, Lørenskog, Norway; 3 National Resource Centre for Women’s Health, Department for Obstetrics and Gynecology, Oslo University Hospital Rikshospitalet, Oslo, Norway; 4 Department for Obstetrics and Gynecology, Innlandet Hospital, Lillehammer, Norway; 5 International School of Public Health, Northern State Medical University, Arkhangelsk, Russia

**Keywords:** Hyperemesis gravidarum, Validity study, Medical Birth Registry of Norway

## Abstract

**Background:**

Valid registration of medical information is essential for the quality of registry-based research. Hyperemesis gravidarum (HG) is characterized by severe nausea and vomiting, weight loss and electrolyte imbalance starting before 22nd gestational week. Given the fact that HG is a generally understudied disease which might have short- and long- term health consequences for mother and child, it is of importance to know whether potential misclassification bias influences the results of future studies. We therefore assessed the validity of the HG-registration in the in Medical Birth Registry of Norway (MBRN) using hospital records.

**Methods:**

The sample comprised all women registered in MBRN with HG and who delivered at Ullevål and Akershus hospitals in 1.1.-31.3.1970, 1.4.-30.6.1986, 1.7.-30.9.1997 and 1.10.-31.12.2001. A random sample of 10 women per HG case, without HG according to MBRN, but who delivered during the same time periods at the same hospitals was also collected. The final sample included 551 women. Sensitivity, specificity, positive and negative predictive values (PPV and NPV) were estimated using strict and less strict diagnostic criteria of HG, indicating severe and mild HG, respectively. Hospital journals were used as gold standard.

**Results:**

Using less strict diagnostic criteria of HG, sensitivity, specificity, PPV and NPV were 83.9% (95% CI: 67.4-92.9), 96.0% (95% CI: 93.9-97.3), 55.3% (95% CI: 41.2-68.6) and 99.0% (95% CI: 97.7-99.6), respectively. For strict diagnostic criteria, being hospitalised due to HG the corresponding values were 64% (95% CI: 38.8-87.2), 92% (95% CI: 90.2-94.6), 18.6% (95% CI: 10.2-31.9) and 99.0% (95% CI: 97.7-99.6).

**Conclusions:**

The results from our study are comparable to previous research on disease registration in MBRN, and show that MBRN can be considered valid for mild HG but not for severe HG.

## Background

During the late 1950s and early 1960s Thalidomide was prescribed to women suffering from nausea and vomiting in early pregnancy, resulting in more than 10 000 limb deformities globally [1,2]. The Medical Birth Registry of Norway (MBRN) was established in 1967 reflecting the need for epidemiological surveillance of birth defects [3]. Since then, MBRN has become a unique source of perinatal data comprising several generations. Additionally, MBRN contains valuable information on maternal diseases before pregnancy, during pregnancy and after pregnancy, although incomplete ascertainment has been and still is a concern [4].

Valid registration of medical information is essential for the quality of registry-based research. Previous studies validating disease registration of in MBRN have shown diverging results, depending on the condition [4-8]. Engeland evaluated the validity of diabetes type 1 among 1.929 million women registered in MBRN using the Norwegian Prescription Database and found a sensitivity of 90% and specificity of 100% [5]. For any type of diabetes the corresponding values were 72% and 99%, although for asthma the sensitivity was 51% and specificity 98%. Smaller studies using hospital records as gold standard have reported the sensitivity for maternal rheumatic disease in MBRN to be 88% and for myasthenia gravis 99% [4,6]. Specificities were not reported. The obstetric sphincter tears registration among 25.761 women in MBRN was evaluated using Patient Administrative Systems finding a sensitivity of 91.8% and specificity of 91.8% [7]. Another study exploring the validity of unexplained foetal deaths among 10.857 singleton pregnancies in MBRN using hospital records and results from autopsies, reported a sensitivity of 78% and specificity of 88% [8].

Hyperemesis gravidarum (HG) is characterised by severe nausea and vomiting starting before the 22nd gestational week, often leading to weight loss and nutritional deficiencies [9,10]. The validity of the HG registration in MBRN has not yet been investigated, although previous studies using the data from MBRN have found the prevalence of HG to be similar to the prevalence observed in neighbouring countries [11-13]. Whereas up to 90% of all pregnant women report nausea or nausea and vomiting (NVP), HG affects 0.3-3.2% [9,13]. Earlier research has been influenced by the fact that HG and the more common NVP have been studied as one and the same condition; but so far we do not know if or how these two conditions are related [14-18].

Additionally HG is a generally understudied disease, which might have short and long term health consequences for mother and child, such as increased risk of rheumatic disease among mothers and increased risk of testicular cancer and leukaemia among children [19-22]. Although MBRN in an international context provides an extremely large dataset, it is important to know whether potential systematic errors influence the results of previous and future studies using MBRN. Our aim was therefore to investigate the validity of the HG registration in MBRN using hospital records as gold standard.

## Methods

Notification to MBRN is compulsory and is provided by midwives and physicians attending the birth using a standardised form [3]. From 1967 to 1998 pregnancy outcomes were notified from the 16th gestational week, after 1998 from the 12th gestational week. Maternal diseases before and during pregnancy are also notified. Women with HG in MBRN were registered according to International Classification of Diagnoses (ICD) [10]. From 1967 to 1998 HG was registered by the ICD-8 codes 638.0 (hyperemesis gravidarum with neuritis), 638.9 (hyperemesis gravidarum without mention of neuritis), and 784. 1 (nausea and vomiting) [11]. From 1999 and onwards HG was registered by the ICD-10 codes O21.0 (mild hyperemesis gravidarum), O21.1 (hyperemesis gravidarum with metabolic disturbances), and O21.9 (vomiting in pregnancy, unspecified) [13].

The sample consisted of all women registered with HG in MBRN who delivered their babies at Ullevål^a^ and Akershus^b^ hospitals during four time periods: 1.1-31.3.1970, 1.4-30.6.1986, 1.7-30.9.1997 and 1.10-31.12.2001. The sample also included a random selection of women not registered with HG in MBRN, but who delivered during the same time periods at the same hospitals; ten women were selected per HG case.

**Figure 1:**
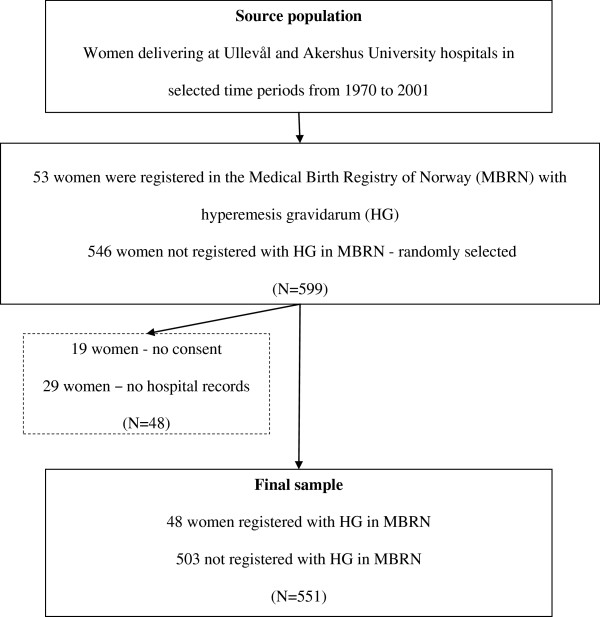
Sampling frame.

Altogether 599 women delivering in Ullevål and Akershus hospitals during the four time periods were selected for the study. Among them, 53 women were registered with HG in MBRN. Informed consent in accordance with guidelines of the Ministry for Health and Care Services was obtained from all women included in the study sample. Altogether, 19 women did not give their consent and were excluded. Furthermore, 29 hospital records were missing resulting in exclusion of these women. Our final study sample therefore comprised 551women among whom 48 women were registered with HG in MBRN and 503 women were not (Figure 1).

The hospital record was considered as gold standard and checked manually. In order to validate the HG registration in MBRN we investigated all information within the women’s hospital records including patient journals, laboratory sheets with test results from blood and urine samples and antenatal cards. An antenatal card is a standardised form all pregnant women in Norway receive at their first routine examination early during the first trimester in pregnancy. The antenatal card is later used as medical record for midwifes and medical doctors during the entire pregnancy. After birth the antenatal card is filed within the hospital record and provides information from all examinations in pregnancy, such as the mother’s height, weight, blood pressure, urine samples, the need of sick leave etc. The antenatal card also provides information on any specific symptom or complaint the pregnant woman might have, such as HG.

In order to be registered with HG based on strict diagnostic criteria, indicating severe HG, the women had to have been admitted to hospital due to HG. In addition two out of the three following symptoms had to be reported in the hospital record; weight loss, dehydration or ketonuria. If the woman had been admitted to hospital because of HG during the actual pregnancy, her patient journal or laboratory sheets provided data on symptoms such as nausea and vomiting, weight loss, dehydration and ketonuria.

In order to be registered with HG based on less strict diagnostic criteria, indicating mild HG, the women did not have to have been admitted to hospital due to HG. If she was not admitted to hospital because of HG, her antenatal card, which is filed within the hospital record, contains the necessary health information. Since most women experience some degree of nausea and vomiting during pregnancy (NVP) we considered that the nausea and vomiting worth describing on her antenatal card or was mentioned in her patient journal would be more pronounced than normal, and therefore should be registered as mild hyperemesis [14]. One hospital record was missing when less strict diagnostic criteria were applied.

Sensitivity, specificity, positive (PPV) and negative (NPV) predictive values were calculated using hospital records as gold standard. Wilson’s method was used to calculate with 95% confidence intervals (CI) for the estimates. All calculations were performed using Confidence Intervals Analysis software.

This study was approved by the Data Inspectorate, the Ministry for Health and Care Services and the Norwegian Scientific-Ethical Committees.

## Results

Sixty-nine percent of the hyperemesis patients were registered by ICD 8 (1967–1998) and 31% by ICD 10 (1999–2006). Among the 48 women with HG in MBRN, 31 women were registered by the ICD 8 code 638.9, 2 women were registered by the ICD 8 code 784.1 and 15 women were registered by the ICD 10 code O21.9.

The results of validating the HG registration in MBRN using hospital records are shown in Table 1 and Table 2. Table 1 reflects the validity of the HG diagnosis in MBRN by the use of strict diagnostic criteria indicating severe HG. Among the 48 women registered with HG in MBRN, 9 women were hospitalised due to severe HG. According to all hospital records, 14 women were hospitalised due to severe HG, indicating that 5 were not registered with HG in MBRN. The use of strict diagnostic criteria resulted in a sensitivity of 64.3% (95% CI: 38.8-83.7), specificity of 92.7% (90.2-94.6), PPV of 18.6% (10.2-31.9) and NPV of 99% (97.7-99.6).

**Table 1:** Validity of HG registration in MBRN using hospital records and strict diagnostic criteria^1^ (N = 551)

	**HG + in hospital record**	**HG – in hospital record**	**Total**
HG + in MBRN	9	39	48
HG – in MBRN	5	498	503
Total	14	537	551
Sensitivity (9/14)	64.3%	95% CI: 38.8-83.7	
Specificity (498/537)	92.7%	95% CI: 90.2-94.6	
Positive predictive value (9/48)	18.6%	95% CI: 10.2-31.9	
Negative predictive value (498/503)	99.0%	95% CI: 97.7-99.6	

^1^ Strict diagnostic criteria indicate severe HG.

**Table 2:** Validity of HG registration in MBRN using hospital records and less strict diagnostic criteria^1^ (N = 550)^2^

	**HG + in hospital record**	**HG – in hospital record**	**Total**
HG + in MBRN	26	21	47
HG – in MBRN	5	498	503
Total	31	519	550
Sensitivity (26/31)	83.9%	95% CI: 67.4-92.9	
Specificity (498/519)	96.0%	95% CI: 93.9-97.3	
Positive predictive value (26/47)	55.3%	95% CI: 41.2-68.6	
Negative predictive value (498/503)	99.0%	95% CI: 97.7-99.6	

^1^ Less strict diagnostic criteria indicate mild HG.

^2^ One medical record went missing during the investigating period.

Table 2 presents the corresponding results applying less strict diagnostic criteria indicating mild HG. Among the 48 women registered with HG in MBRN, 26 had the disease according to the hospital records (Table 2). The use of less strict diagnostic criteria resulted in a sensitivity of 83.9% (95% CI: 67.4-92.9), specificity of 96.0% (93.9-97.3), PPV of 55.3% (41.2-68.6) and NPV of 99.0% (97.7-99.6).

## Discussion

This is the first study to validate the registration of HG in MBRN. When less strict diagnostic criteria, indicating mild HG, were applied, the sensitivity was 83.9%, specificity of 96.0%, PPV of 55.3% and NPV of 95.9%. These results are comparable to previous research on disease registration in MBRN, such as diabetes 1, showing that HG is likewise valid for use in large epidemiologic studies when it comes to the mild form of the disease [4-6]. For strict diagnostic criteria, indicating severe HG, the corresponding figures were 64.3%, 92.7%, 18.6% and 99.0%. The low PPV shows that MBRN is not valid for severe HG. Thirty five percent of the women were admitted to hospital due to HG when less strict diagnostic criteria were used, compared to 100% when strict criteria were used. PPV reflects the probability of having HG in accordance with the hospital record when registered with HG in MBRN, and is depending on the prevalence [13]. Since the registration of rare diseases is influenced by false positive cases, the PPV is expected to be low when the prevalence is low [23].

A major strength of this study is the sample representing all HG patients registered in MBRN among women who delivered during four different time periods from 1970 to 2001 in two large hospitals; hospitals now serving 19% of all births in Norway (unpublished data from MBRN). Although the two hospitals selected might not be representative for all Norwegian hospitals, selection bias is not very likely due to the national guidelines for diagnosing and treating HG worked out by the Norwegian Society for Gynecology and Obstetrics [24]. Furthermore, hospital records are commonly used as “gold standard” when registry data are being validated [4,6,8,25]. A limitation of our study might be the low number of women included.

Errors in information on HG in MBRN might have occurred on three different levels; 1) by the midwife or general practitioner filling in the antenatal card or patient journal, 2) by the midwife or physician attending the birth notifying MBRN, and 3) the registration at MBRN. Even though diagnosing and treating HG in Norway is carried out in accordance with national guidelines worked out by the Norwegian Society for Gynecology and Obstetrics, there is always a chance for misdiagnosing due to inexact diagnostic criteria in clinical practice [26]. For women who had not been hospitalized due to HG, the midwife or physician notifying MBRN had to rely on the information written on the woman’s antenatal card. Although the ICD 8 as well as ICD 10 codes allow differentiating between mild and severe HG, HG is not registered as such in MBRN. In our study MBRN mainly registered HG as 638.9 in ICD 8 and only as O21.9 in ICD 10. Due to the exclusive use of O21.9, describing unspecific vomiting in pregnancy, Kari Klungsøyr who is responsible for their coding procedures in MBRN, was contacted. She confirmed that there might be a potential for misclassification bias during the first period after ICD 10 was introduced (personal communication). Exclusion of O21.9 from the analysis would have resulted in losing a third of the sample, which is why we present our data without excluding those coded as such.

In order to be able to differentiate between mild and severe HG we therefore decided to distinguish between strict and less strict diagnostic criteria, indicating severe and mild HG. The use of the different diagnostic criteria showed that the majority of women registered with HG in MBRN suffered from milder forms and was not hospitalised due to HG. Also the fact that the ICD 8 code 784.1 was included in our study as HG represents a source of error. This is due to the fact that ICD code 784.1 is not related to nausea and vomiting during pregnancy in particular. The reason for including this ICD code in our study, was to be comparable with a previous publication on HG using data from MBRN [11]. When excluding the two women registered with 784.1, the PPV for HG in MBRN increased from 55.3% to 57.8%.

Previous studies validating disease registration in MBRN using hospital records as gold standard did only report sensitivities and can therefore only partly be compared to ours [4,6]. The sensitivity for HG is, however, comparable to the one for rheumatic disease as well as myasthenia gravis [4,6]. Another study used the Norwegian Prescription Database validating the registration of diabetes 1, all types of diabetes, epilepsy and asthma in MBRN [5]. The validity for diabetes 1 in MBRN was in line with our findings for mild HG with a PPV of 56%. The validity for the registration of all types of diabetes, epilepsy and asthma in MBRN was reflected in lower sensitivities, specificities, PPV and NVP than for mild HG. PPV for epilepsy and asthma was 37% and 46%, respectively.

A Swedish study on HG’s effect on pregnancy outcomes suggested that the exposure-outcome associations were diluted due to the probability of registering milder forms of HG since the HG diagnosis not being well defined [27]. Our study shows that this “dilution” is the case for MBRN since MBRN is valid for mild HG. When it comes to differentiating between mild HG and the more common NVP, our sample did not provide the opportunity to investigate this. For future studies it will be important to be able to differentiate between the more common nausea and vomiting in pregnancy, mild HG and severe HG necessitating hospitalisation. Severe forms of HG in particular are found to be associated with adverse pregnancy outcomes, such as preterm birth and low birth weight [28-30]. Severe HG and excessive vomiting in pregnancy has also been reported as risk factors for the development of childhood leukaemia and testicular cancer as well as rheumatic disease among mothers [20,22,31]. In utero exposure to HG has also been linked to a 3.6-fold risk of psychological and behavioural disorders in the offspring [32].

## Conclusions

The results from our validity study show that MBRN may be valid for mild HG, but not for severe HG. Furthermore, the results are comparable to previous research on disease registration in MBRN [4-6]. For future studies on HG, MBRN is valid as database, although the relatively large proportion of false positive cases might influence the exposure-outcome associations in terms of reducing associations closer to the null value.

## Endnotes

^a^Oslo University Hospital Ullevål Hospital since 2009

^b^Akershus University Hospital since 2008

## Abbreviations

HG: Hyperemesis gravidarum; ICD: International Classification of Diseases; MBRN: Medical Birth Registry of Norway; NPV: Negative predictive value; NVP: Nausea and vomiting in pregnancy; PPV: Positive predictive value.

## Competing interests

The authors declare that they have no competing interests.

## Authors’ contributions

AV, AMG and PM designed the study, whereas AV and SL collected the data. AV, PM, SV and AMG all contributed to drafting the manuscript; all involved have approved the final version of the manuscript.

## Pre-publication history

The pre-publication history for this paper can be accessed here:

http://www.biomedcentral.com/1471-2393/12/115/prepub
